# Inhibition of Epithelial CC-Family Chemokine Synthesis by the Synthetic Chalcone DMPF-1 via Disruption of NF-*κ*B Nuclear Translocation and Suppression of Experimental Asthma in Mice

**DOI:** 10.1155/2015/176926

**Published:** 2015-08-02

**Authors:** Revathee Rajajendram, Chau Ling Tham, Mohamad Nadeem Akhtar, Mohd Roslan Sulaiman, Daud Ahmad Israf

**Affiliations:** ^1^Department of Biomedical Science, Faculty of Medicine & Health Sciences, Universiti Putra Malaysia, 43400 Serdang, Selangor, Malaysia; ^2^Institute of Bioscience, Universiti Putra Malaysia, 43400 Serdang, Selangor, Malaysia

## Abstract

Asthma is associated with increased pulmonary inflammation and airway hyperresponsiveness. The interaction between airway epithelium and inflammatory mediators plays a key role in the pathogenesis of asthma. *In vitro* studies evaluated the inhibitory effects of 3-(2,5-dimethoxyphenyl)-1-(5-methylfuran-2-yl)prop-2-en-1-one (DMPF-1), a synthetic chalcone analogue, upon inflammation in the A549 lung epithelial cell line. DMPF-1 selectively inhibited TNF-*α*-stimulated CC chemokine secretion (RANTES, eotaxin-1, and MCP-1) without any effect upon CXC chemokine (GRO-*α* and IL-8) secretion. Western blot analysis further demonstrated that the inhibitory activity resulted from disruption of p65NF-*κ*B nuclear translocation without any effects on the mitogen-activated protein kinase (MAPK) pathway. Treatment of ovalbumin-sensitized and ovalbumin-challenged BALB/c mice with DMPF-1 (0.2–100 mg/kg) demonstrated significant reduction in the secretion and gene expression of CC chemokines (RANTES, eotaxin-1, and MCP-1) and Th2 cytokines (IL-4, IL-5, and IL-13). Furthermore, DMPF-1 treatment inhibited eosinophilia, goblet cell hyperplasia, peripheral blood total IgE, and airway hyperresponsiveness in ovalbumin-sensitized and ovalbumin-challenged mice. In conclusion, these findings demonstrate the potential of DMPF-1, a nonsteroidal compound, as an antiasthmatic agent for further pharmacological evaluation.

## 1. Introduction

Allergic asthma is a chronic inflammatory airway disorder characterized by bronchial hyperreactivity (BHR) to a wide variety of specific and nonspecific stimuli [1]. In most cases, the severity of BHR correlates with the level of airway inflammation, another hallmark of asthma that is associated with eosinophil infiltration, mucus hyperproduction, and increased production of T_H_2 cytokines and allergen-specific IgE [2, 3]. The infiltration of eosinophils, mast cells, and T-lymphocytes into airway epithelia leads to airway inflammation, overproduction of mucus, and airway wall remodeling, which culminates in bronchial hyperreactivity and airway obstruction [4].

The airway epithelium acts as an essential controller of inflammatory, immune, and regenerative responses to allergens, viruses, and environmental pollutants that contribute to asthma pathogenesis [5]. The asthmatic airway epithelium produces chemokines that have prominent effects on leukocyte recruitment and activation. Increasing evidence suggests that the chemokine system coordinates leukocyte recruitment in the pathogenesis of many pulmonary diseases; hence, the accumulation and/or activation of leukocyte populations that drive the asthmatic response could be attenuated by blocking chemokine synthesis or chemokine receptors [6].

Chemokines are small cytokines (8 to 10 kDa) that are primarily involved in attracting and regulating leukocyte trafficking into the tissues, in a process called chemotaxis. To date, more than 40 chemokines have been classified into four subclasses according to their structure: CXC, CC, C, and CX3C. The two main groups are CXC (*α*-chemokines) and CC (*β*-chemokines). CXC chemokines such as IL-8 and growth-regulated oncogene-*α* (GRO-*α*) primarily target neutrophils and have mainly been related to acute inflammatory processes. On the other hand, CC chemokines such as eotaxin, RANTES, MCP-1 to MCP-4, MIP-1*α*, and MIP-1*β* primarily target monocytes, T cells, and eosinophils and are therefore of great relevance in asthma [7, 8].

Tumour necrosis factor-*α* (TNF-*α*) is a proinflammatory cytokine that has been implicated in many aspects of the airway pathology in asthma. TNF-*α* is produced predominantly by macrophages but also other immune cells such as T-lymphocytes, B-lymphocytes, dendritic cells, mast cells, neutrophils, and eosinophils as well as structural cells including fibroblasts, endothelial cells, epithelial cells, and smooth muscle cells. Once released in the airways, TNF-*α* activates the release of multiple inflammatory mediators, chemokines and cytokines, thus promoting ongoing inflammation [9]. Binding of TNF-*α* to its receptor initiates a variety of potential intracellular signaling cascades including activation of MAP kinase pathways (p38, MEK/ERK, and JNK) and proinflammatory transcription factors NF-*κ*B and AP-1, depending on the exact tissue and the expression pattern of associated signaling machinery [10–12].

**Figure 1:**
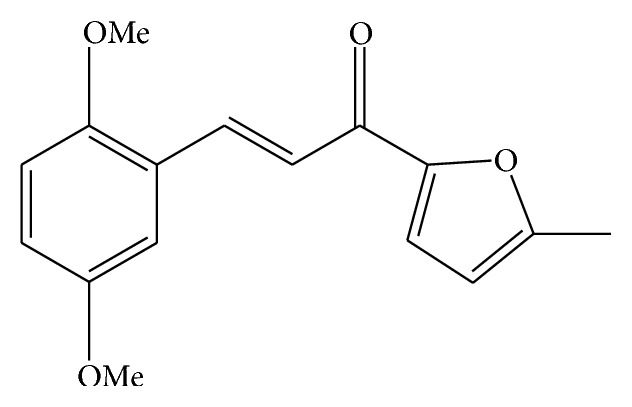
The chemical structure of DMPF-1.

DMPF-1 (3-(2,5-dimethoxyphenyl)-1-(5-methylfuran-2-yl)prop-2-en-1-one; Figure 1) is a synthetic chalcone analogue. Chalcones, which belong to flavonoid family, are made up of two aromatic rings joined by a three-carbon *α*,*β*-unsaturated carbonyl system [13]. Studies have shown that synthetic and naturally occurring chalcones possess a diverse array of pharmacological activities including antimicrobial, antioxidant, anticancer, and anti-inflammatory properties [13, 14]. Furthermore, several chalcone derivatives have also been shown to inhibit airway inflammation and BHR in allergic asthma [15]. We have previously demonstrated that DMPF-1 inhibited nitric oxide production by LPS-stimulated RAW 264.7 murine macrophages [16]. However, the effect of DMPF-1 on airway inflammation has never been studied. Therefore, the objectives of this study were to determine the effect of DMPF-1 upon the synthesis of asthma-related proinflammatory chemokines in TNF-*α*-induced pulmonary epithelial cells and OVA-challenged BALB/c mice. We also attempted to determine the effect of DMPF-1 upon the proinflammatory NF-*κ*B and MAPK signaling pathways in TNF-*α*-induced A549 cells. A final objective in the future involves assessment of the effect of orally administered DMPF-1 upon Th2 cytokine synthesis, eosinophilia, goblet cell hyperplasia, peripheral blood total IgE, and BHR in OVA-challenged BALB/c mice.

## 2. Materials and Methods

### 2.1. Synthesis of DMPF-1

The chalcone derivative DMPF-1 was chemically synthesized at the Institute of Bioscience, Universiti Putra Malaysia, by Claisen-Schmidt condensation reaction. NaOH (2.0 mmol, 40%) was added to a 250 mL single-necked round-bottom flask containing 2-acetyl-5-methylfuran (1.0 mmol) dissolved in methanol (20 mL) and stirred vigorously for 20 minutes in cold water. Then, 2,5-dimethoxybenzaldehyde (1.0 mmol) was added and the reaction mixture was stirred at room temperature for 24 hours. The completion of the reaction was assessed with thin layer chromatography (TLC). The mixture was acidified with concentrated HCl and the reaction mixture was transferred into a separating funnel containing 100 mL distilled water. The yellow layer was extracted with ethyl acetate and the solvent was concentrated in vacuum and dried over anhydrous sodium sulphate. The compound DMPF-1 (Figure 1) was purified by column chromatography using silica gel (100–200 mesh, Merck) and eluted with petroleum ether and ethyl acetate.

### 2.2. Cell Culture

Human A549 lung adenocarcinoma epithelial cells were obtained from the American Type Culture Collection (ATCC). These cells were cultured in RPMI-1640 supplemented with 10% fetal bovine serum (FBS), 4.5 g/L glucose, sodium pyruvate (1 mmol/L), L-glutamine (2 mmol/L), streptomycin (50 *μ*g/mL), and penicillin (50 U/mL) at 37°C in a 5% CO_2_ humidified incubator. The cells were generally maintained to a confluency of 80–90% and detached with trypsin-EDTA. Cell viability was always more than 90%, as determined by trypan blue dye exclusion. The concentration of cells was adjusted to 2 × 10^6^ cells/mL prior to seeding. Cells were stimulated with 10 ng/mL of tumor necrosis factor-alpha (TNF-*α*) (PeproTech, USA) and cotreated with various concentrations of DMPF-1 in all experiments. DMPF-1 was dissolved in 100% dimethylsulfoxide (DMSO) and the final concentration of DMSO was always maintained at 0.1%.

### 2.3. Cell Viability Assay

Cytotoxicity of DMPF-1 was assessed by the MTT cytotoxicity assay. Following treatment with increasing concentrations of DMPF-1 (3.13–100 *μ*M), the cells were incubated at 37°C in a 5% CO_2_ humidified incubator for 24 hours. Then, the cell culture supernatant was removed and MTT solution (5 mg/mL) was added to each well and further incubated at 37°C, 5% CO_2_ for 4 hours. Spent media containing the MTT solution was removed, and the formazan crystals were dissolved in 100 *μ*L/well of DMSO. The absorbance at 550 nm was measured with a microplate reader (VersaMax, Molecular Devices, Sunnyvale, CA, USA).

### 2.4. Chemokine Immunoassay

Following 24-hour coincubation with 10 ng/mL TNF-*α* and increasing concentrations of DMPF-1, spent media was collected and stored at −80°C prior to chemokine immunoassay. The concentrations of MCP-1, IL-8, eotaxin-1 (BD Pharmingen, USA), RANTES, and GRO-*α* (RayBiotech Inc., GA) were quantified with commercially available sandwich ELISA kits. All assays were conducted according to the manufacturer's instructions.

### 2.5. Whole Cell, Nuclear, and Cytoplasmic Protein Extraction

Cells were grown until being confluent in 75 cm^2^ tissue culture flasks. Culture media in the flask was discarded and cells were rinsed twice with ice-cold PBS (pH 7.4) and lysed with lysis buffer (125 mM, 4% SDS, 20% glycerol, 0.004% bromophenol blue, phosphatase inhibitor cocktail, and benzonase nuclease). After a 15 min incubation on ice, cells were scrapped out gently with a cell scrapper and boiled at 90–100°C for 5 min. Cell lysates were left to cool down before being centrifuged at 16000 g, 4°C, for 15 min. The supernatant was collected and stored at −80°C prior to analysis. Protein quantification was performed using the BCA assay kit (Pierce, USA).

Nuclear and cytoplasmic extractions were performed using the NucBuster Protein Extraction Kit (Novagen, CA) according to the manufacturer's instructions. Attached cells in the flasks were rinsed twice with ice-cold PBS (pH 7.4) and lysed with NucBuster Reagent 1. Cells were incubated on ice for 10 min and vortexed at high speed for 30 seconds. Cell lysates were centrifuged at 16,000 g, 4°C for 5 min, and the supernatant was collected as a cytosolic extract. The pellet was resuspended in NucBuster Reagent 2 containing protease inhibitor cocktail and DTT. The supernatant was collected as nuclear extract following centrifugation at 16,000 g, 4°C for 5 min. The concentration of protein in each sample was quantified with a BCA assay kit (Pierce, USA). Both cytosolic and nuclear extracts were stored at −80°C for further analysis.

### 2.6. Western Blot Analysis

Analysis of p38, p-p38, JNK, p-JNK, ERK, and p-ERK proteins was done using whole cell lysates while p65 NF-*κ*B analysis was done on both cytosolic and nuclear extracts. Protein samples (20 *μ*g) were loaded on 10% (for the analysis of p65 NF-*κ*B) and 12% (for the analysis of p38, p-p38, JNK, p-JNK, ERK, and p-ERK) SDS-polyacrylamide gels. Gels were electrophoresed on a Mini-PROTEAN System (BioRad, CA) and blotted onto a PVDF membrane using a Trans-Blot Semi-Dry Transfer Cell (BioRad, CA). The membrane was blocked with 5% BSA for 1 hour prior to overnight incubation at 4°C with rabbit polyclonal antibody specific for p38 (1 : 500), p-p38 (1 : 500), JNK (1 : 500), p-JNK (1 : 500), ERK1 (1 : 1000), ERK2 (1 : 1000), p-ERK (1 : 500), and p65 NF-*κ*B (1 : 1000). After washing three times with TBS-Tween, membranes were hybridized with HRP-conjugated donkey anti-rabbit secondary antibody (1 : 2500–5000) for 2 hours followed by three times washing with TBS-Tween. The same membrane was stripped and reprobed with HRP-conjugated mouse monoclonal antibody specific for *β*-actin (1 : 10,000) or rabbit anti-TFIIB polyclonal antibody (1 : 1000). Membranes were incubated with Super Signal West Femto Maximum Sensitivity Substrate Reagent (Pierce, Rockford, IL) for 5 minutes and bands were viewed under chemiluminescence on a Chemi-Smart gel documentation system (Vilber Lourmet, Marne-la-Vallee, France). Band intensities were quantified with Bio-Profil software (Celbio, Milan, Italy) and normalized by comparison to *β*-actin or TFIIB.

### 2.7. Animals

**Figure 2:**
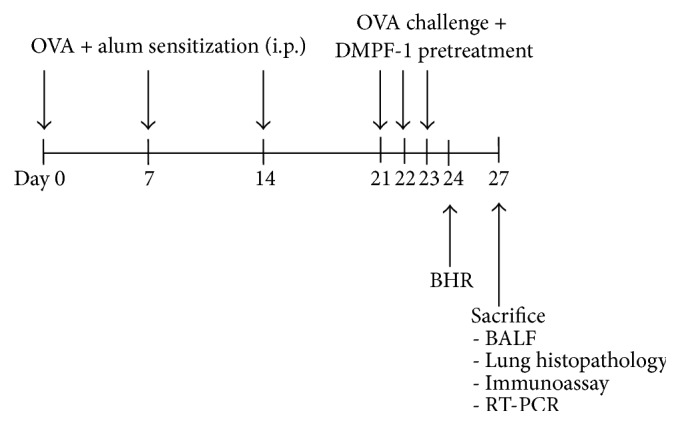
Schematic diagram of the* in vivo* experimental protocol. Mice were sensitized intraperitoneally with OVA (500 *μ*g/mL) and 10% (w/v) alum on days 0, 7, and 14. On days 21, 22, and 23, sensitized mice were pretreated with DMPF-1 intraperitoneally one hour prior to challenge with aerosolized OVA (1%) (w/v). BHR was measured 24 hrs after the last OVA challenge (day 24). The mice were sacrificed on day 27 and BALF, serum, and lung tissues were collected for total and differential cell counts, histopathological evaluation, immunoassay, and RT-PCR.

Female BALB/c mice aged 8–10 weeks were used. The mice were housed at the Physiology Lab Animal Experimentation Room, which was maintained at 22–24°C with a 12-hour dark/light cycle, fed on a commercial lab animal pellet, and provided water* ad libitum*. All experiments were conducted according to protocols approved by the Animal Experimentation Ethics Committee, Faculty of Medicine and Health Sciences, Universiti Putra Malaysia (Figure 2).

### 2.8. Ovalbumin-Induced Experimental Asthma

Mice were sensitized with 500 *μ*g/mL ovalbumin (OVA) and 10% (w/v) aluminium potassium sulphate (alum) (Sigma-Aldrich, MO, USA) in 0.1 mL phosphate buffered saline (PBS). Sensitizations were administered intraperitoneally on days 0, 7, and 14 (weekly intervals). One week following the last sensitization, the mice were challenged with aerosolized OVA (1% (w/v) in PBS) for 30 minutes on three consecutive days (days 21–23). The aerosol exposure was performed in a chamber using an ultrasonic nebuliser (Omron, Japan). Naïve mice were sensitized with OVA and challenged with aerosols of PBS only.

### 2.9. Administration of DMPF-1

Four doses of DMPF-1 (0.2 mg/kg, 2 mg/kg, 20 mg/kg, and 100 mg/kg) were prepared in a vehicle consisting of 90% (v/v) distilled water, 5% (v/v) ethanol, and 5% (v/v) Tween-20. DMPF-1 was given to mice one hour prior to each OVA aerosolization intraperitoneally on days 21, 22, and 23. Mice administered with DMPF-1 appeared healthy, showed regular weight gain and activity levels similar to control mice, and had no ulceration at the injection sites. Dexamethasone (3 mg/kg) was used as a drug control, prepared in the same vehicle, and administrated through the same route. Preliminary studies showed that the vehicle used had no significant effect upon airway inflammation and mediator secretion.

### 2.10. Assessment of Bronchial Hyperresponsiveness (BHR)

Bronchial hyperresponsiveness (BHR) was assessed by methacholine-induced airflow obstruction. BHR was measured at 24 hours (day 24) after the last OVA challenge. Conscious mice were placed unrestrained in a whole body plethysmograph system (Buxco Electronics, Inc., Troy, NY) and challenged with incremental doses of methacholine (6.25, 12.5, 25, and 50 mg/mL) (Sigma-Aldrich, MO, USA) in order to induce BHR. The mice were exposed to each dose of methacholine for 3 min and the readings were taken for 5 min after each nebulization. Bronchopulmonary resistance was expressed as enhanced pause (Penh).

### 2.11. Bronchoalveolar Lavage (BAL) Fluid Collection and Cell Counts

Mice were sacrificed on day 27 (three days after the final challenge dose of OVA). The trachea was cannulated with a 22 G feeding needle and tied with surgical thread. BAL fluid was obtained by flushing both lung lobes with 0.9 mL ice-cold PBS (pH 7.4). Flushing was done four times repeatedly in a slow manner. The bronchoalveolar fluid (BALF) was centrifuged (400 g, 10 min, 4°C) and the supernatant was stored at −80°C prior to quantification of cytokines and chemokines. The cell pellet was resuspended in PBS and cytosmears were prepared on a Hettich centrifuge (500 g, 5 min, 4°C) with cytospin adaptors. Smears were dried overnight and stained with Wright's Stain for differential cell counts. Total cell numbers were counted in a hemocytometer after 1 : 1 dilution of the cell suspension with trypan blue.

### 2.12. Lung Histopathology

Following BALF collection, the lungs and trachea of the mice were fixed with 0.9 mL of 10% formalin through the 22 G feeding needle that was cannulated into the lung. Lungs were then removed and kept in 10% formalin. Lung tissue was fixed in formalin for at least 72 hrs and cut into small pieces prior to tissue processing. The tissue was dehydrated with increasing percentages of ethanol and cleared with xylene on a Leica Automated Tissue Processor TP 1020 (Leica Instrument Gmb, Germany). Sections were embedded in paraffin wax for 24 hours. Embedded tissues were cut into 4 *μ*m sections using a microtome (Leica, IL, USA). Tissue sections were deparaffinized prior to staining with hematoxylin and eosin (H&E) or periodic acid Schiff (PAS). Slides were mounted with DPX mountant and covered with a glass coverslip. The entire slide was scanned initially under low magnification (100x) to count the total number of airways on each slide. All airways on the slide were counted except for those with internal perimeters of more than 500 *µ*m. The range of airway internal perimeters was between 200 *µ*m and 700 *µ*m. Airways with a short/long diameter ratio less than 0.3 were considered as being tangentially cut and excluded in the study. H&E stain was used for the evaluation of cellular infiltration. The number of inflammatory cells in the peribronchial/perivascular regions (within 50 *µ*m from the external perimeter of airways and blood vessels) of each section from the same animal was counted and divided by the number of airways/blood vessels found on each section. The same procedure was repeated on all experimental mice in the same group (*n* = 10) to get the mean number of infiltrated inflammatory cells in the peribronchial/peribronchial region. Three sections were counted for each animal. The numbers of total inflammatory cells per airway and blood vessel were obtained by adding the average number of cells from perivascular count/number of airways and peribronchial count/number of blood vessels. PAS stain was used for histopathological evaluation of goblet cell hyperplasia. The numbers of goblet cells in each airway were counted in a similar manner as mentioned above. To assess goblet cell hyperplasia, the sum of the number of goblet cells was divided by the total number of airways in each slide. The same procedure was repeated on all experimental mice in the same group (*n* = 10) to get the mean number of goblet cells of each group. Three sections were counted for each animal. All the counting in histological studies was carried out in a blinded fashion by two investigators in the laboratory.

### 2.13. Cytokine, Chemokine, and IgE Immunoassay

Concentrations of eotaxin, IL-4, IL-5 (BD Pharmingen, CA, USA), RANTES (RayBiotech Inc., GA), and IL-13 (R&D Systems, Minneapolis, MN) in BALF were quantified using sandwich EIA kits according to the manufacturer's instructions. Similarly, the serum level of total IgE was quantified with a commercially available EIA kit (BD Pharmingen, CA, USA).

### 2.14. Reverse-Transcriptase Polymerase Chain Reaction (RT-PCR)

The total RNA of homogenized lung tissue was extracted using Qiagen RNeasy Plus Mini Extraction kit (Qiagen, USA) according to the manufacturer's instruction. RNA integrity was examined by formaldehyde agarose gel electrophoresis and concentrations were determined by UV spectrophotometry (DU 530 Life Science UV/Visible Spectrophotometer, Fullerton, CA). Master mix was prepared using Qiagen One-Step RT-PCR kit according to the manufacturer's instructions (Qiagen, USA). RNA (2 *µ*g) was added as a template for reverse transcription at 50°C for 30 min, initial PCR activation at 95°C for 2 min, and final extension at 72°C for 10 min in an Eppendorf thermal cycler. PCR products were separated by electrophoresis through a 2% agarose gel, stained with ethidium bromide, and visualized with a gel imaging system under UV light (Vilber Lourmet, Marne-la-Vallée Cedex 1, France). Band intensities were quantified by Bio-Profil software (Celbio, Milan, Italy) and normalized by comparison to the RT-PCR products of glyceraldehyde-3-phosphate dehydrogenase (GAPDH) mRNA. Oligonucleotide primers used for this experiment were published gene sequences.

### 2.15. Statistical Analysis

Statistical analyses were conducted using SPSS version 17.0. One-way ANOVA followed by Dunnett's post hoc test was used to determine statistical significance. Differences were considered to be significant at *P* < 0.05.

## 3. Results

### 3.1. Cell Viability

**Figure 3:**
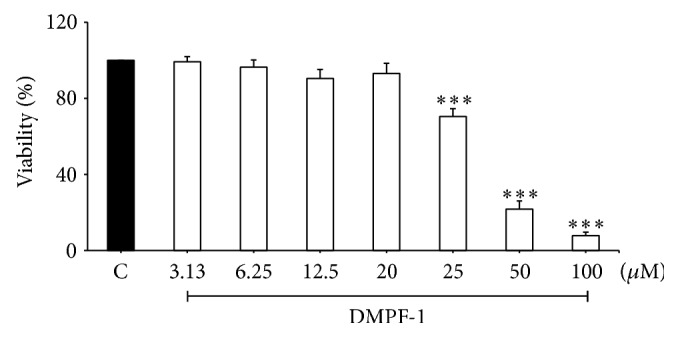
Cell viability of A549 cells following DMPF-1 treatment. Cells were treated with increasing concentrations of DMPF-1 for 24 hours. C stands for vehicle control. The values are expressed as mean ± SEM of three independent experiments performed in triplicate. ^*∗∗∗*^ *P* < 0.005, significantly different from the vehicle control.

An MTT cytotoxicity assay was performed to determine nontoxic concentrations of DMPF-1 to be used in subsequent* in vitro* experiments.  Figure 3 shows that DMPF-1 significantly reduced the viability of A549 cells at 25 *µ*M and above. Thus DMPF-1 was used at 20 *µ*M and below for the subsequent assays.

### 3.2. Chemokine Secretion

**Figure 4:**
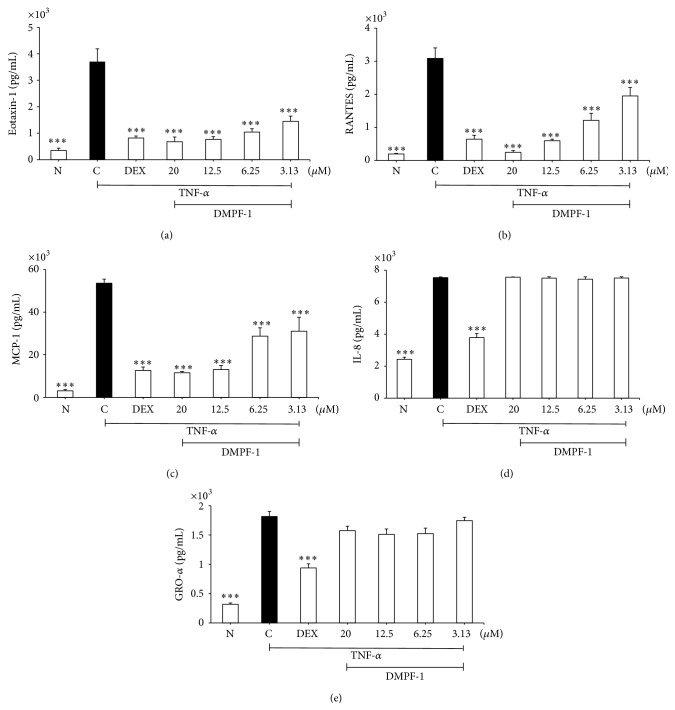
Effect of DMPF-1 on TNF-*α*-induced chemokine secretion by A549 cells. Cells were stimulated with 10 ng/mL of TNF-*α* and treated with increasing concentrations of DMPF-1 for 24 hours and the concentrations of (a) eotaxin-1, (b) RANTES, (c) MCP-1, (d) GRO-*α*, and (e) IL-8 were assayed by EIA. N stands for normal (without TNF-*α*-stimulation); C stands for vehicle control (TNF-*α*-stimulated). The values are expressed as mean ± SEM of three independent experiments performed in triplicate. ^*∗∗∗*^ *P* < 0.005, significantly different from the TNF-*α*-stimulated vehicle control.

Figure 4 shows significant inhibition of eotaxin-1, RANTES, and MCP-1 secretion by TNF-*α*-stimulated A549 cells following DMPF-1 treatment. However, no effect on the secretion of IL-8 and GRO-*α* was observed.

### 3.3. DMPF-1 Disrupts NF-*κ*B but Not MAPK Signaling

**Figure 5:**
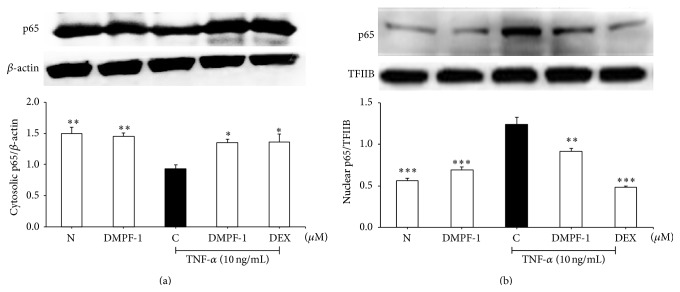
Effect of DMPF-1 on TNF-*α*-stimulated nuclear translocation of NF-*κ*B p65 in A549 cells. Cells were treated with DMPF-1 (7.5 *μ*M) or positive controls in the presence or absence of TNF-*α* for 1 hour. Cytosolic and nuclear fractions were subjected to Western blot analysis. (a) Cytosolic fraction in which protein levels of p65 were normalized to *β*-actin. (b) Nuclear fraction in which protein levels of p65 were normalized to TFIIB. The values are expressed as mean ± SEM of three independent experiments. ^*∗*^ *P* < 0.05, ^*∗*^ *P* < 0.01, and ^*∗∗∗*^ *P* < 0.005, significantly different from the TNF-*α*-stimulated control group.

**Figure 6:**
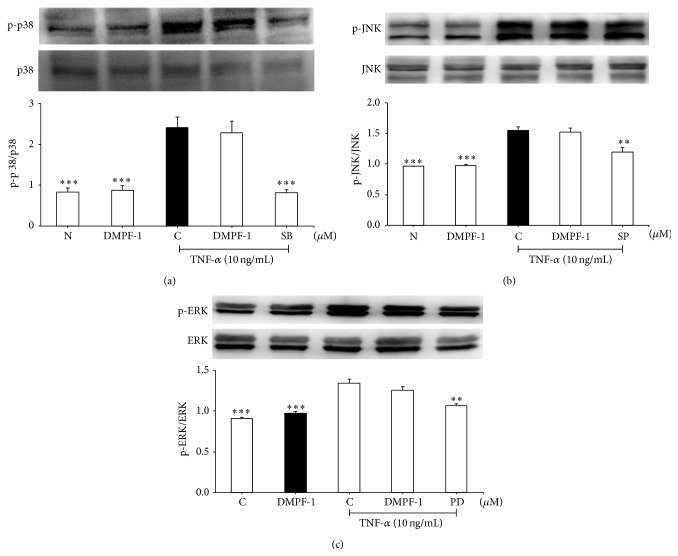
Effect of DMPF-1 on TNF-*α*-stimulated phosphorylation of MAP kinases in A549 cells. Cells were treated with DMPF-1 (7.5 *μ*M) or positive controls in the presence or absence of TNF-*α* for 30 min. Whole cell protein extract was subjected to Western blot analysis. Expression levels of phosphorylated protein of (a) p38, (b) JNK, and (c) ERK were quantified and normalized to nonphosphorylated proteins. The values are expressed as mean ± SEM of three independent experiments. ^*∗∗*^ *P* < 0.01 and ^*∗∗∗*^ *P* < 0.005, significantly different from the TNF-*α*-stimulated control group; SB: SB203580; SP: SP600125; PD: PD98059.

When stimulated with TNF-*α*, p65NF-*κ*B translocates from the cytoplasm into the nucleus (Figure 5). However, treatment with DMPF-1 significantly inhibited the translocation of p65 from cytoplasm into the nucleus.  Figure 6 shows that DMPF-1 had no effect upon the phosphorylation of p38, ERK1/2, and JNK.

### 3.4. DMPF-1 Reduces Bronchial Hyperresponsiveness (BHR)

**Figure 7:**
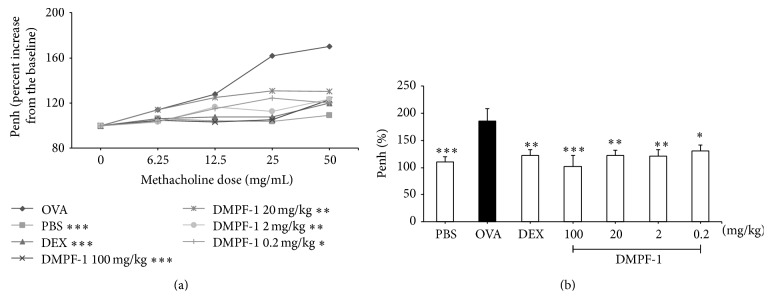
Effect of DMPF-1 on bronchial hyperresponsiveness to methacholine. Female BALB/c mice were sensitized and challenged with OVA 1 hour after treatment with various doses of DMPF-1. After 24 hours of the last OVA challenge, the mice were exposed to (a) increasing doses of methacholine (6.25, 12.5, 25, and 50 mg/mL) in an enclosed chamber for 3 min and the readings were taken for 5 min after each nebulization. (b) The Penh of all groups at the highest dose of methacholine. The values are expressed as mean ± SEM (*n* = 10). ^*∗∗∗*^ *P* < 0.005, significantly different from the OVA-challenged group.

BHR of mice was plotted as percentage increase of enhanced pause (Penh). Methacholine doses were increased from 6.25 mg/mL to 50 mg/mL while PBS was set as a baseline.  Figure 7 shows that exposure to methacholine increased Penh in OVA-sensitized and OVA-challenged mice as opposed to naïve mice (OVA-sensitized and PBS-challenged). DMPF-1 treatment of OVA-challenged mice caused significant reduction of Penh at the highest methacholine dose (50 mg/mL). However, there was no clear dose-response effect among the treatment groups.

### 3.5. DMPF-1 Reduces Total and Differential Cell Counts in BALF

**Figure 8:**
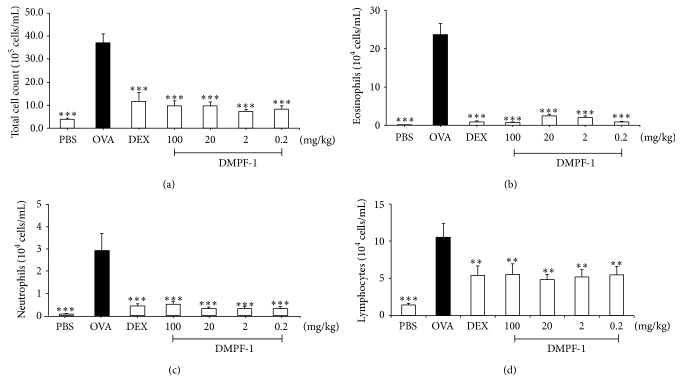
The effect of DMPF-1 on the total and differential cell count in BALF of OVA-sensitized mice. The numbers of (a) total leukocytes and (b) eosinophils, (c) neutrophils, and (d) lymphocytes. The values are expressed as mean ± SEM (*n* = 10). ^*∗∗∗*^ *P* < 0.005, significantly different from the OVA-challenged group.

As shown in Figure 8, there was a significant increment of total cell counts in BALF of OVA-challenged mice compared to naïve mice due to a significant increase in the number of infiltrating eosinophils, neutrophils, and lymphocytes. The number of inflammatory cells recruited to the lung of OVA-challenged mice in response to DMPF-1 was markedly reduced compared to that of the OVA-challenged group. Differential cell counts indicated that the changes in total cell number resulted from an increase in the representation of eosinophils as a proportion of total white blood cells in OVA-challenged mice. Administration of DMPF-1 significantly reduced the influx of inflammatory cells of all three cell types especially eosinophils in BALF in comparison to the OVA-challenged group.

### 3.6. DMPF-1 Reduces Infiltration of Inflammatory Cells into Airways and Goblet Cell Hyperplasia

**Figure 9:**
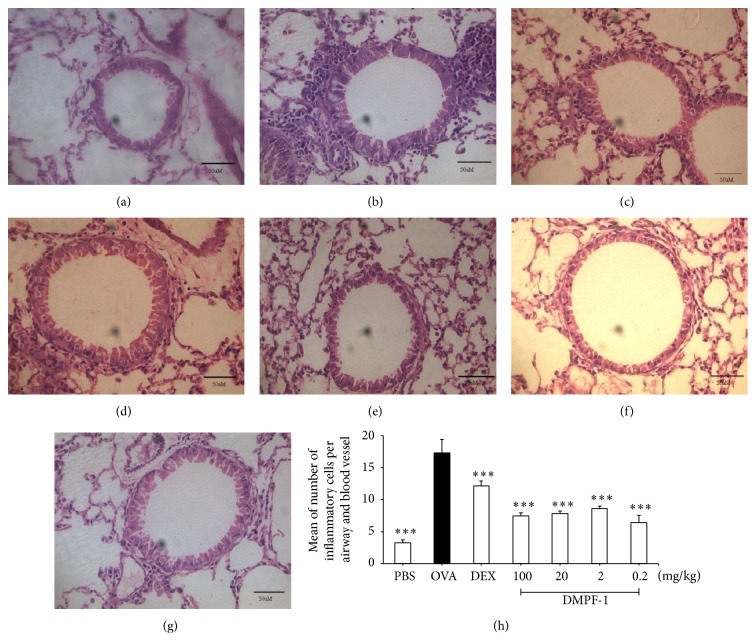
Representative hematoxylin and eosin- (H&E-) stained lung sections. Airways and blood vessels of (a) naïve mice, (b) OVA-challenged mice, (c) dexamethasone-treated mice, (d) 100 mg/kg DMPF-1-treated mice, (e) 20 mg/kg DMPF-1-treated mice, (f) 2 mg/kg DMPF-1-treated mice, and (g) 0.2 mg/kg DMPF-1-treated mice. These photos were taken under 40x objective (400x magnification) using light microscope (Bar = 50 *μ*m). This experiment used 10 mice per group (*n* = 10). (h) For quantitative analysis of infiltration of inflammatory cells, the numbers of total inflammatory cells per airway and blood vessel were obtained by adding the average number of cells from perivascular count/number of airways and peribronchial count/number of blood vessels. The values are expressed as mean ± SEM (*n* = 10). ^*∗∗∗*^ *P* < 0.005, significantly different from the OVA-challenged group.

**Figure 10:**
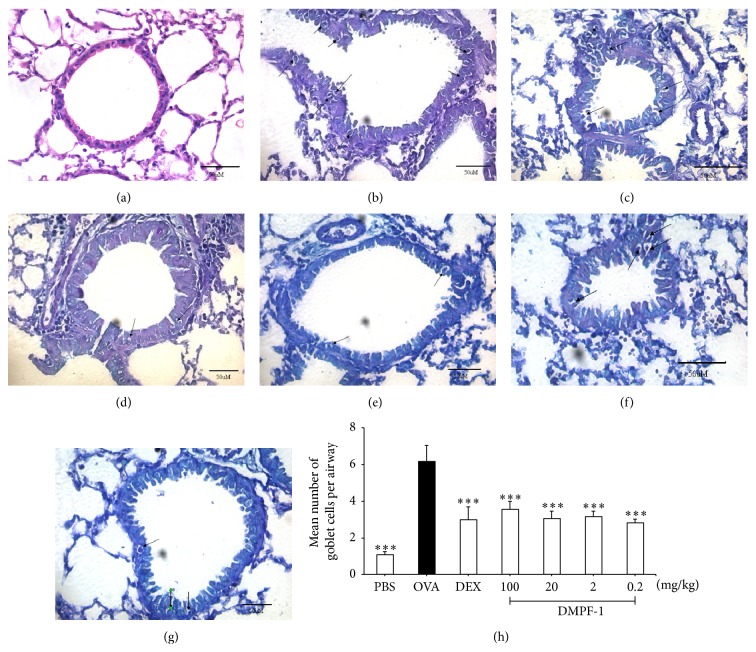
Periodic acid Schiff- (PAS-) stained lung tissue. Airways of mice taken from (a) naïve mice, (b) OVA-challenged mice, (c) dexamethasone-treated mice, (d) 100 mg/kg DMPF-1-treated mice, (e) 20 mg/kg DMPF-1-treated mice, (f) 2 mg/kg DMPF-1-treated mice, and (g) 0.2 mg/kg DMPF-1-treated mice. The goblet cells are indicated by black arrows. These photos were taken under 40x objective (400x magnification) using light microscope (Bar = 50 *μ*m). This experiment used 10 mice per group (*n* = 10). (h) For quantitative analysis of goblet cell hyperplasia, the number of goblet cells in each airway was counted and the sum of the goblet cells was divided by the total number of airways in each slide. The values are expressed as mean ± SEM (*n* = 10). ^*∗∗∗*^ *P* < 0.005, significantly different from the OVA-challenged group.

Inflammatory cell infiltration of the area surrounding the airways and blood vessels in the lung as well as goblet cell hyperplasia was evaluated histologically. Figures 9 and 10 show representative micrographs of both H&E- and PAS-stained sections. In comparison to naïve mice, OVA-sensitized and OVA-challenged mice showed robust pathological changes in allergic pulmonary inflammation which were characterized by extensive infiltration of eosinophils and mononuclear cells around airways and vessels with goblet cell hyperplasia. Mice treated with DMPF-1 demonstrated significant attenuation of pathological changes. To be more specific, H&E-stained sections in Figure 9 show that, in comparison to naïve mice (Figure 9(a)), there were a large number of inflammatory cells concentrated near the airways and in the perivascular and peribronchial areas of OVA-challenged mice (Figure 9(b)). However, the number was markedly decreased following treatment with increasing doses of DMPF-1 (Figures 9(d)–9(g)). Lung histology slides were also stained with PAS to show goblet cells.  Figure 10 shows that OVA-challenged mice (Figure 10(b)) had most PAS-staining goblet cells compared to the naïve mice (Figure 10(a)). Similarly, mice treated with increasing doses of DMPF-1 significantly reduced goblet cell hyperplasia (Figures 10(d)–10(g)).

### 3.7. DMPF-1 Attenuates Excessive Chemokine and Cytokine Synthesis in Lung Tissue and Reduces Total Serum IgE

**Figure 11:**
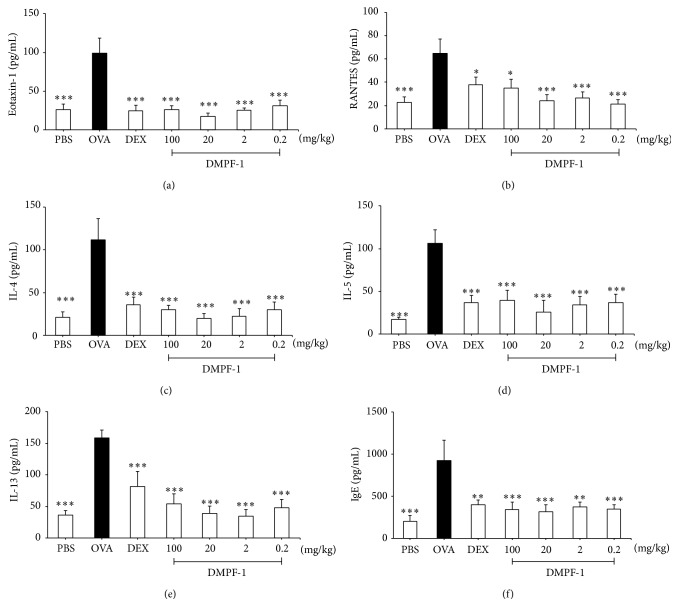
Effect of DMPF-1 on the level of chemokines and Th2 cytokines in the BALF and IgE in the serum of OVA-sensitized mice. After the mice were sacrificed, the BALF was collected and used to measure the level of (a) eotaxin-1, (b) RANTES, (c) IL-4, (d) IL-5, and (e) IL-13 whereas serum from the collected blood was used to measure the (f) IgE level by using ELISA kits according to manufacturer's protocol. The values are expressed as mean ± SEM (*n* = 10). ^*∗*^ *P* < 0.05, ^*∗∗*^ *P* < 0.01, and ^*∗∗∗*^ *P* < 0.005, significantly different from the OVA-challenged group.

**Figure 12:**
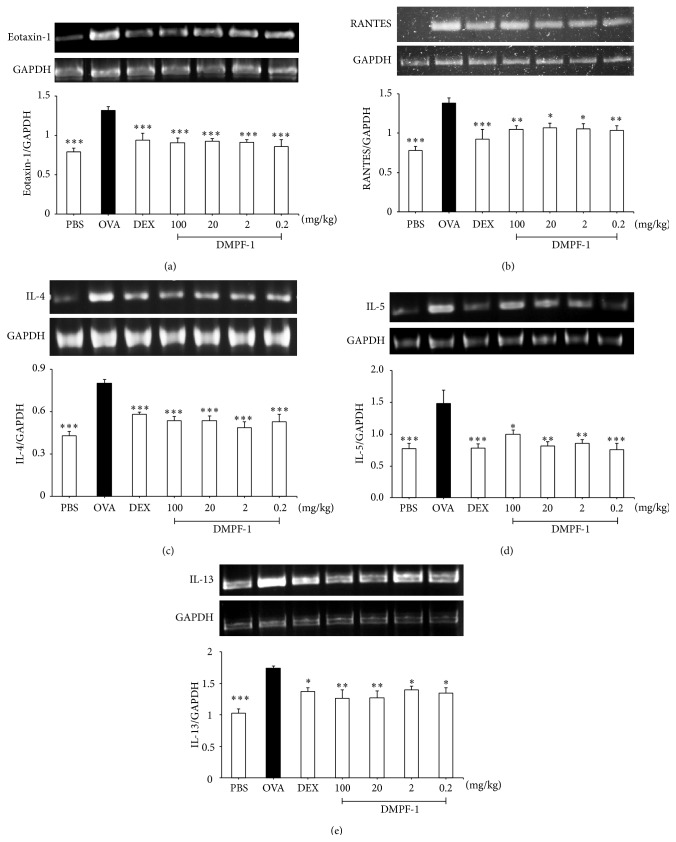
Effect of DMPF-1 on the mRNA level of chemokines and Th2 cytokines on OVA-sensitized mice. Expression levels of mRNA were determined by RT-PCR analysis. After the mice were sacrificed, the lung tissue was collected and the mRNA levels of (a) eotaxin-1, (b) RANTES, (c) IL-4, (d) IL-5, and (e) IL-13 were measured. The values are expressed as mean ± SEM (*n* = 10). ^*∗*^ *P* < 0.05, ^*∗∗*^ *P* < 0.01, and ^*∗∗∗*^ *P* < 0.005, significantly different from the OVA-challenged group.

In comparison to naïve mice, the levels of eotaxin, RANTES, IL-4, IL-5, and IL-13 significantly increased in BALF of OVA-challenged mice (Figures 11(a)–11(e)). However, following treatment with all doses of DMPF-1, the levels of eotaxin, RANTES, IL-4, IL-5, and IL-13 reduced markedly.  Figure 11(f) shows that DMPF-1 had a similar inhibitory effect on IgE level found in the serum of OVA-challenged mice. To further determine whether DMPF-1 modulated eotaxin, RANTES, IL-4, IL-5, and IL-13 in OVA-challenged mice at the transcriptional level, their mRNA levels were determined by RT-PCR. As shown in Figure 12, OVA-challenged group had the highest mRNA expression in comparison to the naïve mice. However, these highly expressed mRNAs were significantly inhibited by all doses of DMPF-1.

## 4. Discussion

Airway inflammation is a dominant feature that leads to clinical symptoms of allergic asthma. The inflammatory response in the asthmatic airways involves a complex interplay of the respiratory epithelium, innate immune system, and adaptive immunity that initiates and drives a chronic inflammatory response. The respiratory epithelium is a major source of many mediators released during airway inflammation in allergic asthma, including Th2 cytokines and chemokines that activate many arms of the immune system [17]. Airway epithelium secretes many chemokines including RANTES, IL-8, eotaxin, and MIP-1*α* to recruit and activate leukocytes, as well as to induce the proliferation and survival of structural cells [18]. Due to the important role of chemokines in airway inflammation, we were interested in determining whether DMPF-1 may alter the synthesis of both CC and CXC chemokines.

Interestingly, DMPF-1 specifically inhibited CC chemokines including RANTES, eotaxin-1, and MCP-1 without inhibiting CXC chemokines such as IL-8 and GRO-*α*. This finding is interesting as CC chemokines have been demonstrated to target monocytes, T cells, and eosinophils that have been shown to have major relevance in the pathogenesis of asthma to CXC chemokines which are mainly related to acute inflammatory processes [19]. The results from Western blot analysis further proved that the specificity of DMPF-1 on CC chemokines may possibly be a result of the selective disruption on NF-*κ*B pathway without affecting the MAPK pathway.

In comparison to the MAPK pathway, the NF-*κ*B pathway indeed plays a more important role in the expression of CC chemokines (MCP-1, eotaxins, and RANTES). Previous studies suggested that the binding of p65 and c-Rel/p65 to the two NF-*κ*B sites (A1 and A2 sites) of the human MCP-1 gene is important in elevating the transcription of this gene [20]. NF-*κ*B-like regulatory sequence can also be found at the eotaxin promoter at position -68 relative to the transcription start site. The presence of NF-*κ*B elements has been shown to be indispensable but not sufficient for TNF-*α*-stimulated chemokine gene expression. Maximal transcriptional activation requires binding sites for additional nuclear factors such as NF-IL-6 and/or SP-1 which are abundant within the eotaxin promoter region and are found to be proximal as well as distal to the eotaxin coding region while recognition sequences for the activator proteins AP-1 and AP-3 or for the phorbol-ester response element PEA3 are only found in more distal eotaxin promoter regions [21]. It was also reported that the RANTES promoter region contains four NF-*κ*B binding sites at positions -30, -44, -213, and -579 relative to the transcription start site. Mutation on any of those NF-*κ*B sites or coexpression of I*κ*B alpha (cytoplasmic inhibitor of NF-*κ*B) markedly reduced the promoter activity and expression of RANTES [22]. Site-directed mutagenesis also indicated that regulation of RANTES promoter activity requires intact NF-*κ*B binding sites while the four putative activator protein-1 (AP-1) recognition sites were dispensable. This finding contradicts that for IL-8, which is a CXC chemokine that requires AP-1 and NF-*κ*B recognition for its full induction of promoter activity by TNF-alpha [23]. AP-1 is a sequence-specific transcription factor composed of members of the Jun and Fos families that mediate gene induction by the phorbol-ester tumor promoter 12-O-tetradecanoylphorbol-13-acetate (TPA).

Three different types of MAPKs (ERK, JNK, and p38) contribute to induction of AP-1 activity through phosphorylation of a different substrate [24]. The less significant role of AP-1 in the expression of MCP-1, RANTES, and eotaxin-1 may explain the selective disruption of DMPF-1 on NF-*κ*B pathway which eventually leads to specific inhibition of CC chemokines but not CXC chemokines.

These encouraging findings prompted us to further determine whether DMPF-1 could inhibit chemokine synthesis in an experimental animal model of allergic asthma. We also sought to determine the effect of DMPF-1 upon other prominent variables of asthma such as increased levels of Th2 cytokines, circulating IgE, airway goblet cell metaplasia, and bronchial hyperresponsiveness [25, 26]. The results from* in vivo* experiments showed that DMPF-1 significantly inhibited the synthesis of Th2 cytokines (IL-4, IL-5, and IL-13) and CC chemokines (eotaxin-1 and RANTES).

Eotaxin and RANTES are produced at high concentrations in asthmatic lungs and are the most important eosinophil chemoattractants in allergic inflammation when acting in synergy with IL-5. RANTES binds many CC chemokine receptors (CCRs), including CCR1, CCR3, and CCR5. On the other hand, eotaxin binds specifically to CCR3, which is highly expressed on eosinophils and has selective chemoattractant activity for eosinophils. In addition, eotaxin induces *α*4- and *β*1-integrin expression on eosinophils, allowing for firm adhesion of eosinophils to the endothelium and transmigration into the site of inflammation [27]. Not only chemokines but also Th2 cytokines such as IL-4 are able to induce eotaxin and are crucial in upregulating the expression of endothelial vascular cell adhesion molecule-1 (VCAM-1) that interacts with very late antigen-4 (VLA-4) to promote the rolling and adhesion of circulating eosinophils to endothelial cells which can then be attracted into target tissues by IL-5 and chemokines [28]. IL-5 induces differentiation and proliferation of bone marrow eosinophils, promotes blood eosinophilia, activates eosinophils, and prolongs their survival [29, 30]. IL-13 is one of the most potent inducers of eotaxin and is another Th2 cytokine that induces VCAM-1 expression on vascular endothelium, activates eosinophils, and promotes their differentiation [31]. All these cytokines can be produced by Th2 cells and detected in BALF of asthmatic subjects and show synergistic effects on the induction of lung eosinophilia. DMPF-1 was demonstrated to significantly inhibit Th2 cytokine and eotaxin synthesis and it reduced eosinophilic infiltration.

The role of Th2 cells in allergic inflammation is not limited to their capacity to promote infiltration of eosinophils and other inflammatory cells to the target tissues. Th2 cells secrete IL-4, IL-5, IL-9, IL-10, and IL-13, which are involved in antibody synthesis [32]. Similarly, chemokines are important in allergy and asthma not only for their role in regulating leukocyte recruitment, but also for their role in regulation of IgE synthesis [33]. The inhibitory effect of DMPF-1 upon the synthesis of both Th2 cytokines and CC chemokines possibly explains the suppression of serum IgE in treated mice.

Bronchial hyperresponsiveness (BHR, defined by exaggerated airflow obstruction in response to bronchoconstrictors), goblet cell hyperplasia, and mucus overproduction are important hallmarks that contribute to airway obstruction in bronchial asthma. IL-13 is responsible for mucus hypersecretion by goblet cells and induces hyperplasia of goblet cells. The resulting hypersecretion of mucus associated with goblet cell hyperplasia causes airway narrowing and thus contributes to airflow obstruction. Together with IL-13, other Th2 cytokines such as IL-4, IL-5, and IL-9 induce mucus hypersecretion through upregulation of goblet cell hyperplasia and contribute to the increase of BHR [34, 35]. The decrease in bronchial hyperresponsiveness following DMPF-1 treatment may be related to inhibition of IL-13 synthesis which has an indirect effect upon mucus hypersecretion.

Although significant effects of DMPF-1 on BHR were noted, we do acknowledge the limitations of noninvasive whole body plethysmography in the assessment of BHR. The use of Penh, a unitless parameter derived mathematically from the respiratory waveform produced by whole-body plethysmography, allows noninvasive and repeated evaluation of airway hyperresponsiveness in unrestrained mice. However, using Penh to evaluate airway resistance is controversial as it may be modified by factors that are not directly related to bronchoconstriction, such as movement, humidity, and temperature as well as upper airway resistance [36–38]. Despite the fact that Penh can be influenced by breathing patterns, many investigators identified a correlative relationship between Penh and airway resistance and suggested that Penh may still be suitable as a preliminary technique [39–41]. Nevertheless there have also been arguments on the validity of invasive methods as this approach requires the need for surgical tracheostomy, thus precluding repeated measurements, the needs for anesthesia, mechanical ventilation, and expertise in handling which may alter baseline breathing patterns. The use of either invasive or noninvasive approaches as the sole indicator of BHR still remains under intense debate. However, in order to gain a more definitive insight of the effect of DMPF-1 on BHR, further investigations on the pharmacology of DMPF-1 will employ both noninvasive and invasive methods.

It is interesting to note that although DMPF-1 demonstrated a dose-dependent inhibitory effect on chemokine synthesis by TNF-*α*-stimulated A549 cells, this was not observed amongst variables measured in our animal model. Obviously dosing regimens cannot be directly translated from cell to animal studies and we would be interested in broadening our dose spectrum in future studies. In conclusion, we have demonstrated that DMPF-1 inhibits the synthesis of CC chemokines* in vitro* and have further confirmed this effect in a standard model of murine allergic asthma. Doses of 0.2 mg/kg and above are effective in alleviating common variables of pulmonary dysfunction in the above model and it is possible that these effects are partly due to inhibition of NF-*κ*B nuclear translocation. Our findings demonstrate that DMPF-1 has potential for further pharmacological evaluation as a new potential nonsteroidal drug lead for the control of asthma.
